# Functional Characterization of Two Novel Mutations in *SCN5A* Associated with Brugada Syndrome Identified in Italian Patients

**DOI:** 10.3390/ijms22126513

**Published:** 2021-06-17

**Authors:** Cristina Balla, Elena Conte, Rita Selvatici, Renè Massimiliano Marsano, Andrea Gerbino, Marianna Farnè, Rikard Blunck, Francesco Vitali, Annarita Armaroli, Alessandro Brieda, Antonella Liantonio, Annamaria De Luca, Alessandra Ferlini, Claudio Rapezzi, Matteo Bertini, Francesca Gualandi, Paola Imbrici

**Affiliations:** 1 Cardiological Center, University of Ferrara, 44121 Ferrara, Italy; bllcst@unife.it (C.B.); francesco.vitali90@gmail.com (F.V.); alessandro.brieda@gmail.com (A.B.); claudio.rapezzi@unife.it (C.R.); doc.matber@gmail.com (M.B.); 2 Department of Pharmacy-Drug Sciences, University of Bari “Aldo Moro”, 70125 Bari, Italy; elena.conte@uniba.it (E.C.); antonella.liantonio@uniba.it (A.L.); annamaria.deluca@uniba.it (A.D.L.); 3 Unit of Medical Genetics, Department of Medical Sciences, University of Ferrara, 44121 Ferrara, Italy; rita.selvatici@unife.it (R.S.); frnmnn1@unife.it (M.F.); annarita.armaroli@unife.it (A.A.); alessandra.ferlini@unife.it (A.F.); 4 Department of Biology, University of Bari “Aldo Moro”, 70125 Bari, Italy; renemassimiliano.marsano@uniba.it; 5 Department of Biosciences, Biotechnologies and Biopharmaceutics, University of Bari “Aldo Moro”, 70125 Bari, Italy; andrea.gerbino@uniba.it; 6 Department of Physics, Université de Montréal, Montréal, QC H3C 3J7, Canada; rikard.blunck@umontreal.ca; 7 Maria Cecilia Hospital, GVM Care & Research, 48033 Cotignola, Italy

**Keywords:** Brugada syndrome, *SCN5A*, electrophysiology, Na^+^ current

## Abstract

Background. Brugada syndrome (BrS) is an autosomal dominantly inherited cardiac disease characterized by “coved type” ST-segment elevation in the right precordial leads, high susceptibility to ventricular arrhythmia and a family history of sudden cardiac death. The *SCN5A* gene, encoding for the cardiac voltage-gated sodium channel Nav1.5, accounts for ~20–30% of BrS cases and is considered clinically relevant. Methods. Here, we describe the clinical findings of two Italian families affected by BrS and provide the functional characterization of two novel *SCN5A* mutations, the missense variant Pro1310Leu and the in-frame insertion Gly1687_Ile1688insGlyArg. Results. Despite being clinically different, both patients have a family history of sudden cardiac death and had history of arrhythmic events. The Pro1310Leu mutation significantly reduced peak sodium current density without affecting channel membrane localization. Changes in the gating properties of expressed Pro1310Leu channel likely account for the loss-of-function phenotype. On the other hand, Gly1687_Ile1688insGlyArg channel, identified in a female patient, yielded a nearly undetectable sodium current. Following mexiletine incubation, the Gly1687_Ile1688insGlyArg channel showed detectable, albeit very small, currents and biophysical properties similar to those of the Nav1.5 wild-type channel. Conclusions. Overall, our results suggest that the degree of loss-of-function shown by the two Nav1.5 mutant channels correlates with the aggressive clinical phenotype of the two probands. This genotype-phenotype correlation is fundamental to set out appropriate therapeutical intervention.

## 1. Introduction

Brugada syndrome (BrS) is an autosomal dominantly inherited cardiac arrhythmia responsible for 4–12% of all sudden cardiac deaths (SCD) in patients without overt structural cardiac abnormalities [1]. The diagnosis is based on the electrocardiogram (ECG) type 1 pattern that is characterized by a distinct coved-type ST-segment elevation with negative T wave in the right precordial leads. Sodium channel blockers are used as additional diagnostic tools to unmask asymptomatic patients and are contraindicated in BrS [2]. Syncope or SCD often occur during rest or sleep and are usually due to polymorphic ventricular tachycardia (VT), which can degenerate in some patients into ventricular fibrillation (VF). BrS occurs predominantly in males of >40 years of age with a male:female ratio of 9:1 [3].

To date, mutations in 25 different genes have been linked to BrS, 18 of which encoding ion channel subunits and 7 encoding regulatory proteins, including *SCN5A*, *SCN10A*, *SCN1B*, *PKP2*, *RANGRF*, *TRPM4*, and several calcium and potassium channels genes [4,5,6]. In about 20–30% of BrS probands, mutations have been found in *SCN5A*, encoding for the cardiac voltage-gated sodium channel Nav1.5 [1]. Nav1.5 consists of a pore-forming α-subunit composed of four homologous domains (DI–IV), each containing six transmembrane segments (S1–S6), with S1–S4 forming the voltage sensor domain (VS) and the S5 and S6 the pore module (PM) [7]. Most *SCN5A* mutations are missense, are localized to the transmembrane segments of the channel and cause different degrees of loss-of-function (LoF) either by impairing trafficking or by modifying Nav1.5 gating properties [8,9,10,11,12,13,14]. LoF can be worsened at higher temperatures for some *SCN5A* mutations, accordingly with the trigger effect of fever in affected patients [15]. Implantable cardioverter defibrillator (ICD) is the first line therapy in BrS high-risk patients; the ablation of the right ventricular outflow tract is recently gaining consideration [16]. Quinidine, a class Ia antiarrhythmic drug, and isoprenaline, a β adrenoreceptor agonist, have been proven useful in patients with contraindication for ICD and on suppression of VT/VF in some BrS patients [17].

One major concern in the pathogenesis and management of BrS is that patients can present with an array of clinical manifestations, ranging from asymptomatic (the vast majority) to high-risk clinical course, even within the same family. Complex genotypes and inheritance patterns have been described in BrS patients, probably accounting for the reported variable expressivity [18]. Many of the mutations identified in other genes have been found in single families and are only responsible for 5% of the BrS cases; therefore, their role in BrS remains to be investigated [6]. *SCN5A* mutations are, however, likely responsible for a minority of cases and many patients lack a genetic diagnosis. Digenic inheritance has also been reported, which underlines mutation load as a pathogenic basis for BrS [19]. Thus, whereas in some families BrS can be considered a monogenic disease, in others this disease has an oligogenic origin and BrS phenotypes likely result from the interplay of rare and common multiple variants and can partially overlap those of a cardiomyopathy [20,21]. The poor genotype–phenotype correlation and the lack of validated predictors of cardiac risk make the therapeutic management of BrS difficult, especially for asymptomatic patients and those at low risk (drug-induced patients).

In this setting, the identification of a *SCN5A* mutation, family segregation analysis and functional studies can contribute to prognostic risk stratification and provide additional information to clinic to address the proper management of affected families [22].

Here, we describe in depths two Italian BrS families carrying two novel mutations in *SCN5A*, Pro1310Leu, and Gly1687_Ile1688insGlyArg, and provide the functional characterization of mutant channel to understand the likelihood of pathogenicity and draw a genotype-phenotype association.

## 2. Results

### 2.1. Clinical and Genetic Analysis

We identified two novel *SCN5A* mutations in Italian BrS patients. The missense variation c.3929C>T (Pro1310Leu, named P1310L throughout the text) in *SCN5A* exon 22 in patient I and the in-frame insertion c.5058_5059insGGCCGC (Gly1687_Ile1688insGlyArg, named Ins1687GR throughout the text) in *SCN5A* exon 28 in patient II. Both variants are novel and not found in gnomAD exomes and genomes (MAF = 0). In silico prediction tools define P1310L as likely pathogenic and the Ins1687GR insertion as of uncertain significance.

**Figure 1:**
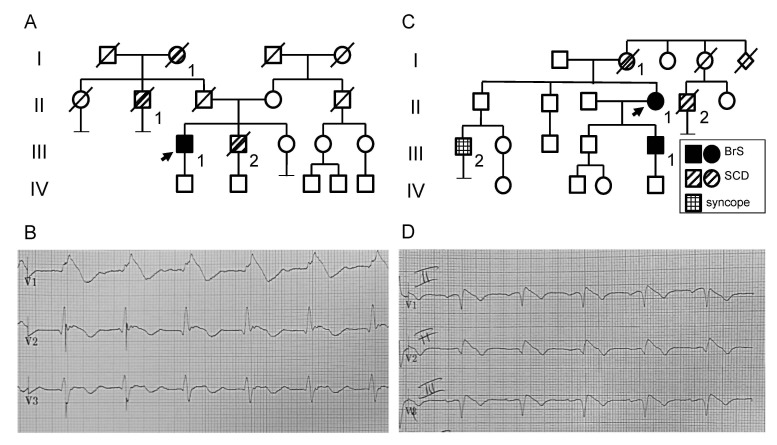
Pedigree and ECG of the BrS families. (**A**) Pedigree of family of patient I (III-1) carrying the missense variation c.3929C>T (Pro1310Leu) in *SCN5A*. The genetic analysis was performed only in proband’s son who resulted negative for the Pro1310Leu variation. I-1: SCD at 87 years; II-1: SCD at 78 years; III-2: SCD at 51 years. (**B**) ECG of patient I shows Brugada type 1 pattern in V1-V2 recorded at standard intercostal space. (**C**) Pedigree of family of patient II (II-1) carrying Gly1687_Ile1688insGlyArg in heterozygosis in *SCN5A*. Her son (III-1) carries both the maternal variation and the His558Arg benign polymorphism in *SCN5A*. The Ins1687GR insertion was searched exclusively in the two subjects manifesting symptoms and/or BrS ECG pattern, in light of the uncertain significance of the variation. I-1: SCD at 38 years; II-2: SCD at 50 years; III-2: syncope and negative drug-induced testing for BrS. (**D**) ECG of patient II shows Brugada type 1 pattern in V1-V2 recorded at second intercostal space.

Patient I. The missense variant P1310L was identified in a 64 years-old man who presented a type I Brugada pattern on ECG at the age of 56 (III-1; Figure 1A,B). He had a positive family history because the proband’s brother died suddenly at 51 years of age (Figure 1A). He received an ICD in primary prevention of SCD. During the follow up, he developed multiple repetitive ventricular arrhythmias treated by the ICD. Due to the recurrent arrhythmias, he underwent a successful epicardial radiofrequency ablation. Asymptomatic cases from family I did not perform the genetic analysis due to non-compliance, except for proband’s son who resulted negative for the P1310L variation.

Patient II. In family II, the in-frame six nucleotides insertion Ins1687GR was searched exclusively in the two subjects manifesting symptoms and/or BrS ECG, considering the uncertain significance of the variation at the time of genetic counselling. The Ins1687GR insertion was identified in a 57 years-old female resuscitated from cardiac arrest at the age of 28 during post-partum (II-1; Figure 1C,D). She received an ICD in secondary prevention of SCD. The proband’s mother died suddenly at the age of 38 years. The mutation segregates in the 36 years-old proband’s son who showed a spontaneous type 1 BrS pattern. He did not have any symptom or arrhythmic event and received an ICD in primary prevention. The proband’s son also carries the His558Arg benign polymorphism in *SCN5A* [23].

### 2.2. Functional Characterization of P1310L Mutant Channels

**Figure 2:**
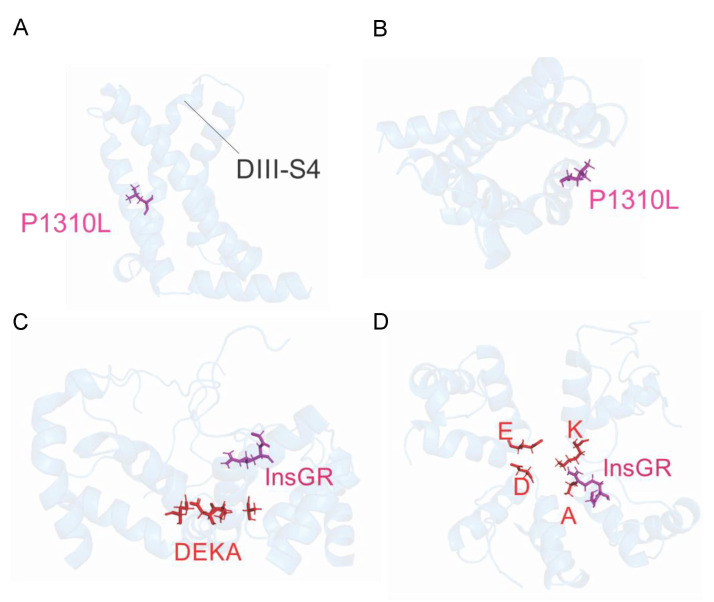
Localization of the BrS mutations in the Nav1.5 channel modeled on the 3D structure of the rat Nav1.5 α-subunit (pdb: 6UZ3). Lateral (**A**) and top (**B**) view of Nav1.5 channel showing the P1310L mutation depicted in purple. Lateral (**C**) and top (**D**) view of the Nav1.5 channel showing the Ins1687GR insertion in purple. The DEKA residues are shown in red.

We generated a model of human Nav1.5 built upon the 3D structure of the recently determined cryo-electron microscopy structure of rat Nav1.5 α-subunit [24]. P1310L consists in the substitution of a proline with a leucine at S4 in DIII of the Nav1.5 channel, a position that is conserved within the family of Nav1.x channels (Figure 2A,B and Figure S1) [7,24].

**Figure 3:**
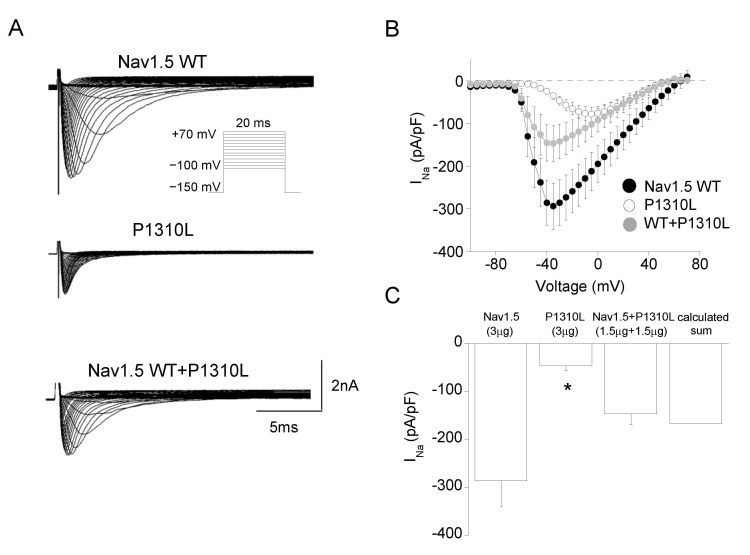
Current density of Nav1.5 WT, P1310L and WT+P1310L channels expressed in HEK 293 cells. (**A**) Representative sodium current traces from cells transfected with Nav1.5 WT (3 µg), P1310L (3 µg), and Nav1.5+P1310L (1.5 µg +1.5 µg) cDNAs. The voltage protocol is shown in the inset. (**B**) IV plot showing the mean current density of Nav1.5 WT, P1310L and Nav1.5+P1310L channels as a function of membrane potential. (**C**) Bar graph showing the mean current density measured at −30mV for the indicated channels. Data are mean ± SE; *n* = 11–20 cells. * *p* < 0.05 for P1310L channels compared with Nav1.5 WT.

To test whether P1310L mutation was responsible for BrS in the affected patient, we recorded sodium currents through the patch clamp technique from HEK 293 cells expressing either Nav1.5 WT or mutant channels. Figure 3A shows representative sodium currents elicited by WT and P1310L channels. Typical WT sodium currents start to activate at −60 mV, peak at −45 mV and decrease in amplitude at more depolarized potentials due to a reduction in driving force. As shown in the IV plot in Figure 3B, peak current density was significantly reduced by more than 3-fold in P1310L channels compared with WT (Table 1).

**Figure 4:**
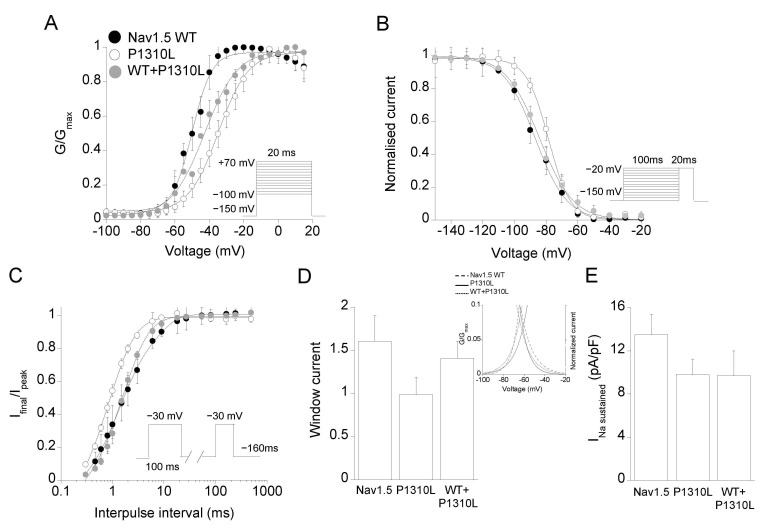
Biophysical properties of Nav1.5 WT, P1310L, and WT+P1310L channels expressed in HEK 293 cells. (**A**) Voltage-dependence of activation. (**B**) Voltage-dependence of fast inactivation. (**C**) Recovery from fast inactivation. Voltage protocols are indicated in the insets and detailed in the Supplementary Materials and Methods. (**D**) Bar graph showing window current measure for the indicated channels. The inset shows the triangular area under the overlapping point of the activation and inactivation curves of the respective channels. (**E**) Bar graph showing the amplitude of the sustained current measured at the end of a 100 ms pulse at −30 mV for the indicated channels. Data are mean ± SE; *n* = 6–20 cells.

We then determined whether modifications of the voltage-dependent activation and inactivation by P1310L mutation might contribute to the reduced activity of P1310L channels and be of pathogenic relevance for BrS. As shown in Figure 4A, the midpoint activation voltage for P1310L channels was shifted by +15 mV towards positive potentials with respect to WT, increasing the degree of depolarization required for activation (Table 1). The voltage-dependence of fast inactivation was instead slightly shifted by +7 mV towards more positive voltages for P1310L channels relative to WT (Figure 4B). P1310L channels also showed a faster recovery from fast inactivation with respect to WT (Figure 4C; Table 1). Current decay measured at −30 mV was instead not modified by the mutation (Table 1).

The small positive shift in the voltage-dependence of fast inactivation and accelerated recovery from inactivation would argue for an increase in P1310L channel availability leading to a mixed phenotype. Actually, among the voltage sensor mutations identified to date, the very close R1309H mutation, was associated with an overlapping phenotype [25]. We then determined whether P1310L may affect sustained and window currents. The analysis of the area beneath the intersection of activation and steady-state inactivation curves demonstrated that P1310L decreased the amplitude of the window current by 40% compared with Nav1.5 WT (Figure 4D). P1310L also reduced the density of the sustained current measured at −30 mV (Figure 4E; Table 1).

Nav1.5 sodium channels can form dimers upon direct interaction between α-subunits, which suggests the possibility that dominant-negative effect exerted by mutant on WT subunit may occur in the heterozygous patient [26,27]. The co-expression of equal amount of WT and P1310L cDNAs gave rise to sodium currents that were similar to the calculated sum of those carried by WT and mutant expressed alone (Figure 3C). In addition, the analyses of the biophysical properties measured for the currents evoked by WT+P1310L channels suggested a predominant LoF defect for WT+P1310L channels compared with Nav1.5 WT (Figure 4A–E).

### 2.3. Functional Characterization of Ins1687GR Mutant Channels

**Figure 5:**
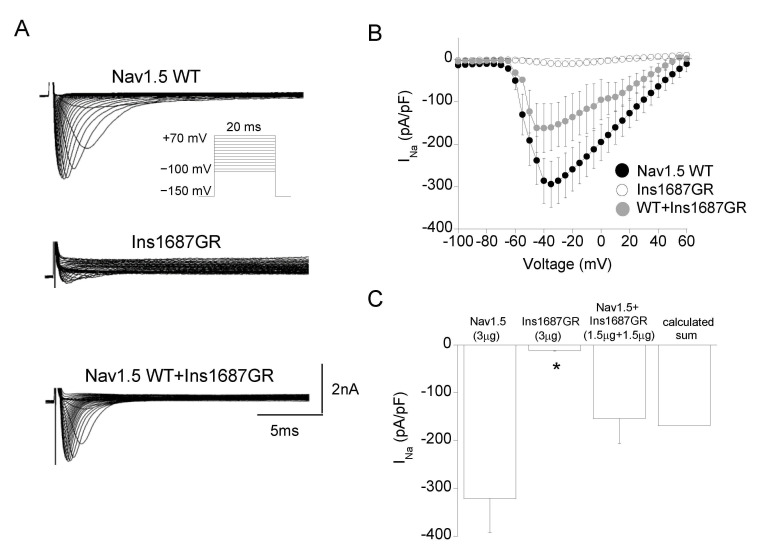
Current density of Nav1.5WT, Ins1687GR, and WT+Ins1687GR channels expressed in HEK 293 cells. (**A**) Representative sodium current traces from cells transfected with Nav1.5 WT (3 µg), Ins1687GR (3 µg) and Nav1.5+Ins1687GR (1.5 µg +1.5 µg) cDNAs. The voltage protocol is shown in the inset. (**B**) IV plot showing the mean current density of Nav1.5 WT, Ins1687GR and WT+Ins1687GR channels as a function of membrane potential. (**C**) Bar graph showing the mean current density measured at -30mV for the indicated channels. Data are mean ± SE; *n* = 11–21 cells. * *p* < 0.05 for Ins1687GR channels compared with Nav1.5 WT.

**Figure 6:**
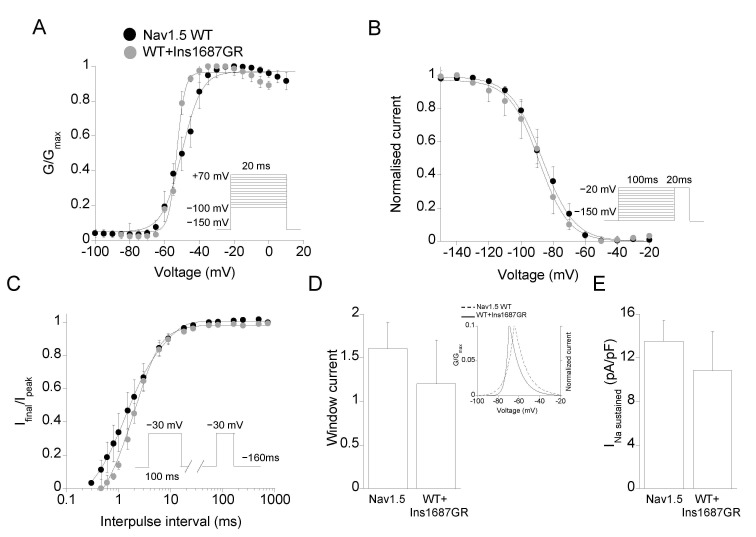
Biophysical properties of Nav1.5WT and WT+Ins1687GR channels expressed in HEK 293 cells. (**A**) Voltage-dependence of activation. (**B**) Voltage-dependence of fast inactivation. (**C**) Recovery from fast inactivation. Voltage protocols are indicated in the insets and detailed in the Supplementary Materials and Methods. (**D**) Bar graph showing window current measure for the indicated channels. The inset shows the triangular area under the overlapping point of the activation and inactivation curves of the respective channels. (**E**) Bar graph showing the amplitude of the sustained current measured at the end of a 100 ms pulse at −30 mV for the indicated channels. Data are mean ± SE; *n* = 6–20 cells.

The mutation Ins1687GR consists in two amino acids, Gly and Arg, being inserted at position 1687 of the S5-P loop of DIV of the Nav1.5 channel. As for P1310L, Ins1687GR mutation occurs at a conserved and functionally relevant pore region within the family of Nav1.x channels (Figure 2C,D and Figure S1) [7,24]. Figure 5A shows representative current traces recorded from cells expressing Ins1687GR channels. Ins1687GR insertion caused a complete loss of Nav1.5 function reducing the sodium current density by more than 25-fold (Figure 5A,B). Unfortunately, the very small current level recorded from Ins1687GR channels hampered any further biophysical analysis of the mutant channel alone. The co-expression of equal amount of WT and Ins1687GR cDNAs gave rise to sodium currents that were similar to the calculated sum of those carried by WT and mutant expressed alone (Figure 5C) and with biophysical properties similar to those of Nav1.5 WT (Figure 6A–E; Table 1).

### 2.4. Cellular Localization of Nav1.5 WT, P1310L and Ins1687GR Mutant Channels

**Figure 7:**
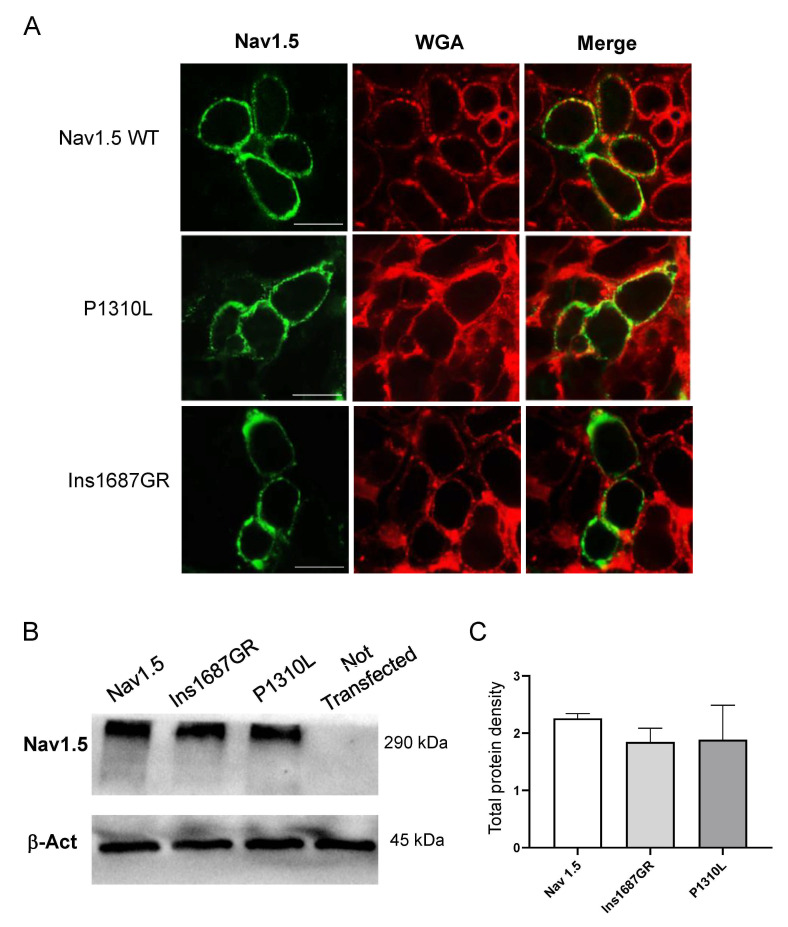
Plasma membrane localization of Nav1.5 WT, P1310L and Ins1687GR channels. (**A**) Immunofluorescence confocal microscopy analysis of the Nav1.5 WT, P1310L and Ins1687GR channels (green signal; left) and plasma membrane marker WGA-555 (red signal, middle) in HEK 293 cells grown on coverslips. The overlay column reports the co-localization of the two fluorescence signals (yellow, right). WGA, Wheat Germ Agglutinin. The horizontal bar indicates 10 μm. (**B**) Immunoblotting analysis of Nav1.5 WT, Ins1687GR and P1310L channels expressed in HEK 293 cells. The position of molecular weight markers is at the right of the blots. Expressions of β-actin are displayed as controls for the loaded protein amounts. (**C**) Bar graph showing the total protein expression level for the indicated channel. For total protein expression, density was standardized as the ratio of the Nav1.5 signal to the cognate β-actin signal. Quantitative analysis was performed from 3 independent experiments.

Reduction in current density could be due to dysfunctional channel gating or impaired trafficking of an otherwise functional channel or both. We therefore performed confocal microscopy analysis to assess the impact of P1310L and Ins1687GR mutations on the localization of Nav1.5 channels in HEK 293 cells. We used wheat germ agglutinin (WGA) AlexaFluor™555 to stain cells plasma membrane and evaluated the co-localization with the anti-Nav fluorescence. As shown in Figure 7A, Nav1.5 WT was mainly expressed at the plasma membrane as shown by the high degree of co-localization with WGA-555. Despite producing smaller sodium currents, P1310L showed membrane expression very similar to that of Nav1.5 WT channels (Figure 7A). In addition, the total protein amount was similar for WT and P1310L channels expressed in HEK 293 cells (Figure 7B,C). Similarly, Ins1687GR channels appeared correctly inserted at the plasma membrane and quantitative analysis revealed similar total protein expression level for Ins1687GR and WT (Figure 7A–C).

### 2.5. Effect of Mexiletine Incubation on Nav1.5 WT and Ins1687GR Mutant Channels

**Figure 8:**
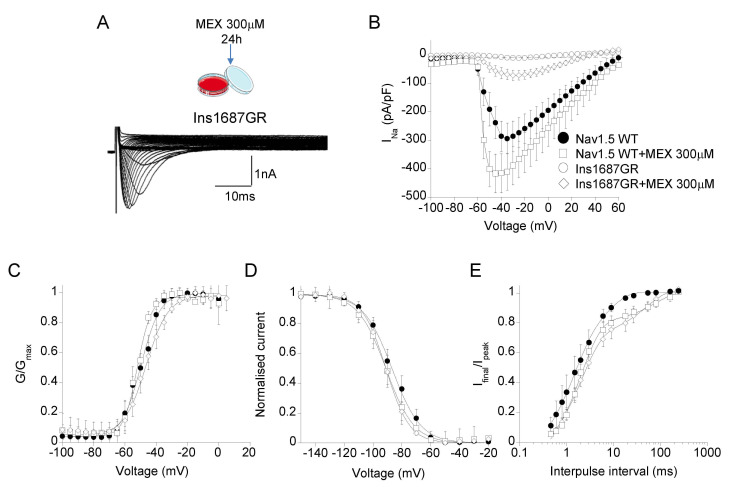
Effect of 24h incubation with 300 μM mexiletine on Nav1.5 WT and Ins1687GR channels. (**A**) Representative sodium current traces from cells expressing Nav1.5 WT and Ins1687GR channels in control solution and after 24 h incubation with 300 μM mexiletine. Mexiletine, was washed out prior to recording sodium currents. (**B**) IV plot showing the mean current density of Nav1.5 WT and Ins1687GR channels as a function of membrane potential before and after 300 μM mexiletine incubation. (**C**) Voltage-dependence of activation for Nav1.5 WT and Ins1687GR channels in control solution and after 300 μM mexiletine incubation. (**D**) Voltage-dependence of fast inactivation for Nav1.5 WT and Ins1687GR channels in control solution and after 300 μM mexiletine incubation. (**E**) Recovery from fast inactivation for Nav1.5 WT and Ins1687GR channels before and after 300 μM mexiletine incubation. Data are mean ± SE; *n* = 6–8 cells.

Mexiletine is a class Ia antiarrhythmic drug that reversibly blocks Nav1.5 channels by binding to a local anesthetic binding site including the S6 DIV [7]. Incubation with high concentrations of mexiletine has been shown to increase membrane expression and current of both Nav1.5 WT and some BrS mutated channels [28,29]. Thus, to increase the membrane expression and current amplitude of Ins1687GR mutant channels, we incubated HEK 293 cells expressing Nav1.5 WT and Ins1687GR channels for 24 h with 300 μM mexiletine. The drug was washed out prior to recording sodium currents. A total of 300 μM mexiletine caused a ~30% increase of Nav1.5 WT current density and did not affect activation and inactivation properties (Figure 8B–E; Table 2). Similarly, mexiletine 300 μM increased the current amplitude of Ins1687GR channels by 7-fold compared with that of non-treated channels (Figure 8B; Table 2). This allowed the evaluation of the biophysics of mutant channels. No significant change was observed in the activation and fast inactivation properties of mutant channels with respect to Nav1.5 WT channels (Table 2).

## 3. Discussion

### 3.1. Genotype-Phenotype Correlation

Mutations in *SCN5A*, encoding for the cardiac voltage-gated sodium channel Nav1.5, represent a frequent cause of BrS and, for this reason, S*CN5A* screening commonly follows clinical diagnosis in affected patients [6]. However, *SCN5A* is a highly polymorphic gene and 2-5% of healthy subjects of different ethnicity carry missense *SCN5A* variants of unknown significance [30]. This adds to the variable expressivity and incomplete penetrance reported for BrS patients, that, even in the presence of a *SCN5A* mutation, may challenge the causative effect of the identified genetic variant. Thus, despite discriminating between pathogenic mutations and harmless variants is of critical importance for the therapeutic management of genotype-positive BrS patients, the interpretation of the genetic test results is often hard. In this context, functional in vitro studies, addressing the impact of the mutation on channel function, represent a powerful tool for clarifying pathogenicity and risk stratification and for exploring pharmacological approaches based on mutations defects.

Here, we describe the functional characterization of two previously unreported *SCN5A* mutations occurring in BrS families, classified as with an uncertain pathogenic role by in silico tools. Our results confirm that loss of Nav1.5 activity is one of the molecular mechanisms underlying BrS symptoms and agree with the observation that the diagnosis of *SCN5A* mutations is associated with an elevated risk of major arrhythmic events in both Asian and Caucasian populations [17,31,32]. More specifically, P1310L and WT+P1310L channels showed reduced macroscopic current density due to altered channel gating, being P1310L channel membrane expression preserved. The positively shifted voltage-dependent activation of both P1310L and WT+P1310L channels appears to principally accounts for the reduced open probability of the channels at physiological potentials and correlates with the position of the P1310L mutation at S4 in DIII, an important region for the voltage-sensitive gating of the channel (Figure 2A,B). As clarified by the recently solved 3D structure of rat Nav1.5 (pdb: 6UZ3) [24], during depolarization, the VS of the four domains moves the sliding helix S4 quickly outward to activate the channel and this voltage-dependent conformational change is transferred to the pore domain through the S4-S5 linker. Fast inactivation, occurring in cardiac channels within a few milliseconds of their activation, is promoted by the interaction of an isoleucine–phenylalanine–methionine (IFM) motif, located in the DIII-DIV linker, with amino acid residues in the S4-S5 linkers in DIII and DIV and in the intracellular ends of the S5 and S6 segments of DIV. The replacement of a rigid, hydrogen-bond acceptor proline with the branched-chain amino acid leucine at position 1310 of S4 DIII, between R1309 and R1312, might alter both the conformational changes required for the voltage sensor movement and the normal coupling of activation to fast inactivation [24]. The LoF defect of the channel is in agreement with the BrS phenotype of the proband who received an ICD in primary prevention and during follow up developed an arrhythmic storm treated with epicardial transcatheter ablation.

The insertion of the two amino acids glycine and arginine at the highly conserved pore region (DIV P loop; [24]) of the Nav1.5 channels reduced the sodium current density without affecting the amount of protein expressed at the cell membrane, with respect to WT. In agreement with previous findings, mexiletine was able to increase, albeit modestly, sodium currents carried by Ins1687GR channels, which seem to activate and fast inactivate similarly to Nav1.5 WT. The Ins1687GR insertion places the positively charged arginine side chain at the entrance of the electronegative ion conduction pathway in DIV through an apparently structurally normal Nav1.5 protein (Figure 2C,D). In turn, we could assume a reduction of sodium conductance due to a lower local Na^+^ concentration at the pore entry. Other pore mutations associated with BrS have been shown to drastically impact Nav1.5 channel function and a similar hypothesis has been postulated for the mutations T1711R in DIV and G1422R in DIII [11,12,13,14,24]. In a very close site, the D1690N mutation, identified in a BrS patient, produced a similar marked reduction in sodium current density [23]. Despite the notion that women are somehow protected from BrS and are less represented in the BrS population, the severe biophysical profile displayed by the pore Ins1687GR mutation may give reason to the clinical phenotype shown by the female carrier who had a major arrhythmic event during post-partum and lost her mother prematurely. Published data suggest that sex hormones might play a role in the phenotypic manifestations of BrS, although the basis for gender-related differences is not yet fully understood [33]. The Ins1687GR mutation segregates in the proband’s son, who also presents the *SCN5A* H558R variant and received an ICD in primary prevention. The benign H558R polymorphism is present in 20% of the population and has been shown to be protective against the BrS phenotype [34,35]. Whether the presence of the additional *SCN5A* polymorphism might contribute to the asymptomatic clinical course of the proband’s son remains to be established.

### 3.2. Pathophysiology

Nav1.5 channels form different macromolecular complexes at different microdomains in cardiomyocytes and carry the inward current responsible for the main component of the initial upstroke of the action potential [36,37]. The loss of inward current and consequent increase of outward potassium current may generate a “transmural dispersion of repolarization” between right ventricle epicardium and endocardium, which can facilitate and explain reentry-based arrhythmias [38,39]. Alternatively, the reduction of sodium current, by impairing phase 0 of the action potential, may slow the electrical conduction through the right ventricle outflow tract, causing action potential re-entry and abnormal heart rhythm [38,39]. In addition, it is now increasingly recognized that alterations in Nav1.5 may affect both ionic and non-ionic processes that alone or in concert contribute significantly to arrhythmogenesis and mechanical dysfunction and likely explain the BrS spectrum of clinical phenotypes [40,41]. These cellular processes, including an altered interaction with neighboring proteins, developing with different features in different patients, may also contribute to the clinical characteristics of Ins1687GR and P1310L families.

In conclusion, our results confirm the pathogenicity of two novel mutations identified in families with BrS and major arrhythmic events. The identification and functional assessment of novel *SCN5A* variants in cell lines and further exploration of the multifunctional effects of Nav1.5 in patients’ iPSCs-derived cardiomyocytes, are essential steps for understanding BrS pathogenesis, for a better risk stratification of BrS patients and for developing personalized therapeutic interventions [42].

## 4. Materials and Methods

### 4.1. Clinical and Genetic Analysis

Patients with spontaneous type I BrS pattern with or without previous symptoms were evaluated at the Cardiogenetic Clinic—University Hospital S. Anna—Ferrara. All patients underwent standard 12 leads ECG with V1-V2 at 2nd and 3rd intercostal space, Holter monitoring, and two-dimensional and three-dimensional echocardiography. Written informed consent for genetic testing was obtained (local ethical committee approval 26/7/2012, P. 7/2012). DNA was isolated from the peripheral blood by standard methods.20 NGS analysis was performed using a custom panel in patient I (Pro1310Leu) and in the proband’s son. A commercial gene panel PED MASTR Plus (Agilent, Santa Clara, CA, USA) was used for patient II and for the proband’s son (Gly1687_Ile1688insGlyArg) [19]. Runs were performed on a MiSeq-Dx sequencer. Data were analyzed and filtered using Sophia Genetics DDM software (https://dropgen.sophiagenetics.com; licence from 9 January 2018).

### 4.2. Mutagenesis and Nav1.5 Channel Expression

Mutations were introduced into the plasmid pRcCMV-hNav1.5 using the QuickchangeTM site-directed mutagenesis kit (Agilent, Santa Clara, CA, USA) [43]. HEK 293 cells were transiently transfected in 100-mm dishes with WT (3 μg) or mutant (3 μg) pRcCMV-hNav1.5 and pCD8-IRES-hβ1 (1.5 μg) expressing the sodium channel auxiliary β1 subunit and the CD8 receptor gene reporter, using the calcium phosphate precipitation method. The complete coding region of the cDNA was sequenced to exclude polymerase errors. The transfected cells were identified by microbeads coated with anti-CD8 antibodies (Dynabeads M-450 CD8; Dynal, Great Neck, NY, USA) and were used for electrophysiological recordings. For co-expression experiments, equal amount of WT and mutant channels cDNAs (1.5 μg + 1.5 μg) were transfected together with pCD8-IRES-hβ1 (1.5 μg) and currents compared with those generated by transfecting Nav1.5 WT cDNA alone (3 μg).

### 4.3. Electrophysiology

Whole-cell sodium currents were recorded at room temperature from HEK 293 cells 48 h after transfection using an Axopatch 200A amplifier, a Digidata 1550B digitizer, and the pClamp 10.6 software (Molecular Devices, San Jose, CA, USA). Current signals were filtered at 5 kHz and sampled at 10 kHz. Patch-clamp pipettes were pulled from borosilicate glass using a vertical puller (Narishige, London, UK) to a resistance of 3–4 MΩ. The internal solution contained (in mM): NaCl 10, CsF 120, CsCl 10, EGTA/CsOH 5, HEPES 5 (pH 7.2 with CsOH). The external solution contained (in mM): NaCl 150, KCl 4, CaCl_2_ 2, MgCl_2_ 1, glucose 5, HEPES 5 (pH 7.4 with NaOH) [43]. Detailed protocols are described in Supplementary Materials and Methods.

### 4.4. Homology Modeling

A homology model of Nav1.5 was built from the crystal structure of the ratNav1.5 (PDB 6UZ3) [25]. Residues that differed or were missing in the ratNav1.5 as compared with the human Nav1.5 were replaced and modeled, respectively, using Modeller 9.25.49,50. We introduced the mutations P1310L and Ins1687GR in the respective domain in the Nav1.5 α-subunit. The resulting channel was introduced into a POPE: POPC: PSPS membrane (3:2:1) using CHARMM-GUI11-13 and was equilibrated using NAMD 2.14.14 [44,45].

### 4.5. Immunocytochemistry

HEK 293 cells were grown on glass coverslips, transfected with cDNAs encoding Nav1.5 WT or mutant channels and β1 subunit, and subjected to immunofluorescence 48h after transfection. Transfection was performed using Lipofectamine™ 2000 reagent (ThermoFisher Scientific, Waltham, MA, USA). Transfected HEK 293 cells were fixed and permeabilized in PFA with 0.1% Triton X-100 for 20 min at room temperature. After washes with PBS, cells were blocked in saturation buffer (1% bovine serum albumin in PBS) for 30 min at room temperature (RT) and incubated with a mouse monoclonal anti-sodium channel pan primary antibody (1:1000; S8809; Sigma-Aldrich Merck Life Science, Milano, Italy) for 2 h at RT in blocking buffer. After three washes in PBS cells were incubated with 488 Alexafluor-conjugated secondary antibodies (ThermoFisher Scientific, Waltham, MA, USA) for 1 h at RT. Plasma membrane was stained with wheat germ agglutinin, Alexa Fluor™ 555 Conjugate (WGA-555; ThermoFisher Scientific, Waltham, MA, USA) in Hank’s balanced salt solution (HBSS) for 30 min at 37 °C at a concentration of 5 µg/mL. Cell coverslips were mounted on glass microscopy slides and observed on a Nikon Eclipse TE 2000-U fluorescent microscope equipped with a 40X/1.30 N.A. fluor objective (Nikon Corporation, Tokyo, Japan) and a spinning-disk confocal setup (Crisel Instruments, Rome, Italy). Nav1.5 fluorescence was excited with a green laser (SVL-473-0200 at 473 nm of excitation wavelength) and recorded at 520 nm emission wavelength. Wheat germ agglutinin AlexaFluor™ 555 fluorescence was excited using a mercury lamp light, selecting an excitation at 555 nm on the excitation filter wheel and emission at 585 nm on the emission filter wheel. Excitation light was projected through 1000 pinholes (Ø 70 µm) using a CREST CARVII™ spinning disk. Images were collected by a Photometrics Cool Snap HQ camera (1392 × 1040 imaging pixels) and digitalized with the MetaMorph^®^ software (Molecular Devices, San Jose, CA, USA).

### 4.6. Western Blot Analysis

To measure total Nav1.5 protein expression, HEK 293 cells were transfected with Nav1.5 WT and mutants cDNAs. 24h after transfection, cells were harvested in 200 μL of cold RIPA buffer (20 mM Tris-HCl, 150 mM NaCl, 1.5% Non-idet P-40, 100 mM sodium orthovanadate and a protease inhibitor cocktail) and placed for 10 min in ice. To complete cell lysis, suspensions were passed through a syringe with a needle for 10 times. After 15 min in ice, cell lysates were centrifuged at 14,000 rpm for 30 min at 4 °C and supernatant was collected. Total protein amounts were quantified by using a BCA protein assay kit (Bio-Rad, Hercules, CA, USA). Total proteins were separated on a 7.5% SDSPAGE and transferred onto nitrocellulose membrane for 1 h at 200 mA (SemiDry transferblot, Bio-Rad Laboratories, Segrate (MI), Italy). Membrane was blocked for 1 h with 0.2 M Tris-HCl, 1.5 M NaCl and pH 7.4 buffer (TBS) containing 5% non-fat dry milk and 0.5% Tween-20 and was incubated overnight at 4 °C with a mouse monoclonal anti-sodium channel pan primary antibody (S8809; Sigma-Aldrich Merck Life Science, Milano, Italy) diluted 1:400 and monoclonal mouse anti-Actin (Sc-47778, Santa Cruz Biotechnology, Dallas, TX, USA) diluted 1:300 with TBS containing 5% non-fat dry milk. After three washes with TBS containing 0.5% Tween-20 (TTBS), membrane was incubated for 1 h with goat anti-mouse IgG conjugated to horseradish peroxidase (Biorad Laboratories, Segrate (MI), Italy). Membrane was then washed with TTBS, developed with a chemiluminescent substrate (Clarity Western ECL Substrate; Bio-Rad Laboratories, Segrate (MI), Italy), and visualized on a Chemidoc imaging system (Bio-Rad Laboratories, Segrate (MI), Italy). Western blots were quantified with Image Lab software (Bio-Rad Laboratories, Segrate (MI), Italy), which allows the chemiluminescence detection of each experimental protein band to obtain the absolute signal intensity automatically adjusted by subtracting the local background.

### 4.7. Statistical Analysis

Statistical analysis was performed using Student’s *t*-test, with *p* < 0.05 or less considered as significant. Results are reported as mean ± SEM from the indicated number of cells.

## Figures and Tables

**Table 1:** Current density and biophysical parameters of Nav1.5 WT and BrS mutant P1310L and Ins1687GR channels.

Channel Type	Peak Current Density	Voltage Dependent Activation		Voltage Dependent Fast Inactivation		Time Constant of Inactivation	Recovery from Inactivation		Sustained Current Density
	−30 mV, pA/pF	V_h_, mV	k, mV	V_h_, mV	k, mV	−30 mV, ms	τ_fast_, ms (A1%)	τ_slow_, ms (A2%)	−30 mV, 100 ms, pA/pF
**Nav1.5 WT**	−286 ± 53 *n* = 11	−49.6 ± 0.6 *n* = 6	6.0 ± 0.4	−86.7 ± 0.5 *n* = 18	9.9 ± 0.4	0.86 ± 0.07 *n* = 14	1.2 ± 0.1 (87%) *n* = 12	6.7 ± 1.1 (13%)	13.7 ± 1.9 *n* = 20
**P1310L**	−46 ± 10 * n = 20	−34.7 ± 0.6 * n = 19	8.7 ± 0.6	−78.5 ± 0.4 * n = 15	7.0 ± 0.3	1.02 ± 0.09 *n* = 18	0.78 ± 0.2 * (78%) *n* = 8	2.4 ± 1.2 * (22%)	9.8 ± 1.5 *n* = 16
**WT+** **P1310L**	−146 ± 23 *n* = 9	−42.0 ± 0.5 *n* = 9	9.2 ± 0.8	−84.0 ± 0.6 *n* = 15	10.0 ± 1.0	1.06 ± 0.17 *n* = 11	1.5 ± 0.6 (83%) *n* = 10	4.7 ± 1.2 (17%)	9.7 ± 2.4 *n* = 13
**Ins1687GR**	−11.1 ± 1.5 * n = 21	/	/	/	/	/	/	/	/
**WT+** **Ins1687GR**	−153 ± 52 *n* = 7	−52.5 ± 0.4 *n* = 6	5.1 ± 0.3	−88.0 ± 1.0 *n* = 6	9.3 ± 0.7	0.99 ± 0.12 *n* = 6	1.6 ± 0.4 (88%) *n* = 6	8.0 ± 3.1 (12%)	10.8 ± 3.6 *n* = 6

* *p* < 0.05 for mutant channels compared with Nav1.5 WT.

**Table 2:** Current density and biophysical parameters of Nav1.5 WT and BrS mutant Ins1687GR channels expressed in HEK 293 cells after 24 h incubation with mexiletine 300 μM.

Channel Type + Mexiletine 300 µM	Current Density	Voltage Dependent Activation		Voltage Dependent Inactivation		Time Constant of Inactivation	Recovery from Inactivation	
	−30 mV, pA/pF	V_h_, mV	k, mV	V_h_, mV	k, mV	−30 mV, ms	τ_fast_, ms (A1%)	τ_slow_, ms (A2%)
**Nav1.5 WT**	−388 ± 78 *n* = 6	−51.9 ± 0.4 *n* = 6	4.4 ± 0.3	−91.0 ± 0.5 *n* = 6	9.7 ± 0.4	1.17 ± 0.13 *n* = 6	2.1 ± 0.1 (83%) *n* = 6	22 ± 4 (17%)
**Ins1687GR**	−72 ± 16 * n = 8	−49.7 ± 0.5 *n* = 6	6.0 ± 0.5	−91.0 ± 0.4 *n* = 7	8.2 ± 0.4	1.20 ± 0.20 *n* = 6	1.99 ± 0.1 (73%) *n* = 6	20 ± 4 (27%)

* *p* < 0.05 for mutant channels compared with Nav1.5 WT.

## Data Availability

The data presented in this study are available on request from the corresponding author. Genetic data have been submitted to LOVD at https://databases.lovd.nl/.

## Supplementary Materials

The following are available online at https://www.mdpi.com/article/10.3390/ijms22126513/s1, Supplementary Materials and Methods; Figure S1: Amino acids alignment of Nav1.x proteins highlighting the position of the P1310L and Ins1687GR BrS mutations; Figure S2: (A) Immunoblotting analysis of Nav1.5 WT, Ins1687GR and P1310L channels expressed in HEK 293 cells as in Figure 7B. The positions of molecular weight markers are at the right of the blots. Expressions of β-actin are displayed as controls for the loaded protein amounts. (B) Stain-free blot image, corresponding to the total protein load, of the immunoblot reported in (A); Supplementary References.
