# Supplementary material for: Functional Characterization of Two Novel Mutations in SCN5A Associated with Brugada Syndrome Identified in Italian Patients
Source: Int J Mol Sci. 2021 Jun 17;22(12):6513. doi: 10.3390/ijms22126513 (PMC8234720; doi:10.3390/ijms22126513)
Supplement: Supplementary file 1 [file ijms-22-06513-s001.zip › ijms-1239008-supplementary.pdf]

International Journal of *Molecular Sciences*

MDPI

Supplementary Materials

Supplementary Materials and Methods

Clinical and genetic analysis

PED MASTR Plus detects Primary Electrical Disorders and includes 51 genes. The Exome Variant Server (ESP), the Exome Aggregation Consortium (ExAC) and the gnomAD database with a frequency greater than 0.1% were used to filter out common variants. Potential disease-causing missense variants were assessed using Mutation Taster, Polyphen2 and SIFT. Varsome (https://varsome.com/) was used as a tool to sum up actual knowledge about the variants. Identified variants were classified according to American College of Medical Genetics and Genomics (ACMG) guidelines [1]. The molecular confirmation of variants was performed by standard Sanger sequencing on an automated analyzer (ABI PRISM ® 3130). *KCNQ1* exon 9 and *SCN2B* exon 4 not included in the PED assay were analyzed by Sanger sequencing. The same was done for regions with depth of coverage less than 20X for both panels. The utilized accession number for defining *SCN5A* mutations is NM_198056.2.

Electrophysiology

Recordings were generally initiated 5 min after establishment of whole-cell configuration. Only cells with access resistance <5 MΩ were used and input resistances were typically >600MΩ. The capacitance currents and series resistances (80%) were partially compensated using the amplifier circuit. Leak currents were always <200 pA at the holding potential (−150 mV). Cells exhibiting peak current amplitudes <500 or >5000 pA were excluded from analyses. Patch clamp recordings were analyzed off-line by using ClampFit 10.6 (Molecular Devices, San Jose, CA, USA) and Kaleidagraph Software (Synergy Software, Reading, PA, USA).

To determine the current-voltage relationships, currents were elicited by 20-ms depolarizing pulses from −100 to +70 mV in 5-mV increments from a holding potential of −150 mV (5-s interpulse duration). Peak current amplitudes at each voltage step were defined as the maximal current amplitudes. Current amplitudes were normalized to the cell capacitance (Cm) to obtain current density (pA/pF).

The voltage-dependence of activation was measured using the same protocol. To analyze the voltage dependence of activation properties, peak current amplitudes at each voltage were converted to conductances (*G*) as follows:

$G_{Na} = I_{Na}/(V_m - E_{rev})$, where Vm is the voltage clamp step and E_{rev} the reversal potential for sodium ions which was determined from Nernst equation (E_{rev}=+68mV). Values of G_{Na} were normalized to the maximum conductance and plotted as a function of voltage. Conductance-voltage relationships were fitted with the Boltzmann equation:

$G/G_{max} = 1/(1 + \exp(-(V_m - V_h)/k))$, in which V_h is the membrane potential for half-maximum activation and k is the slope factor [2-4].

The voltage dependence of steady-state inactivation was measured using a standard two-pulse protocol where a 100-ms conditioning prepulse to membrane potentials between −150 and -20mV (to induce steady-state inactivation) in 10-mV increments, was followed by a 20-ms test pulse to −20mV, from a holding potential of −150 mV, (5-s interpulse duration). Current amplitudes measured at each test pulse were normalized to the maximal current amplitude (I_{max}) and plotted as a function of prepulse voltage. The steady-state inactivation curves were fitted with the Boltzmann equation:

$I/I_{max} = 1/(1 + \exp((V_m - V_h)/k))$, in which V_h is the membrane potential for half-inactivation and k is the slope factor [2-4].

Sodium current decay at -30mV was fitted with a single exponential function to calculate the inactivation time constant τ, using equation:

$I(t) = I_0 + A \times \exp(-t / \tau)$ where A is the amplitude and τ is the decay time constant.

The sustained INa current was assessed during a 100-ms depolarizing voltage steps from −120 mV to −30 mV.

Recovery from inactivation was assessed using a standard two-pulse protocol (5-s interpulse duration) to −30 mV from a holding potential of −160 mV. The voltage between the pulses was -160 mV and interpulse interval was varied between 0.3 to 1000 ms. Peak current amplitudes measured at each test pulse were normalized to current amplitudes measured during each prepulse and plotted against interpulse intervals. The time course of recovery from inactivation was analyzed by fitting the current amplitudes ratio (I_{final}/I_{peak}) with the double exponential function:

$I(t) = [A_{fast} \times \exp(t/\tau_{fast})] + [A_{slow} \times \exp(t/\tau_{slow})]$,

with τ_{fast} and τ_{slow} respectively the fast and slow recovery time constants and A_{slow} and A_{fast} the amplitudes of the slow and fast components [2-4].

To analyze the effect of the mutation on the Nav1.5 window current, the area delimited by the intersection of activation and fast inactivation voltage-dependence curves was calculated using the trapezoidal rule (Sigma Plot 8.02, Systat Software Inc., San Jose, CA, USA). The overlap of the two curves defines a window of potentials (range of voltages) at which the channels have a small probability of being partially activated but not fully inactivated and where a fraction of channels may open [5]. A larger window current predicts an increase in the fraction of channels that open at hyperpolarized voltages resulting in an increase in inward Na^+ current.

All the chemicals were purchased from Sigma-Aldrich Merck Life Science (Sigma-Aldrich Merck Life Science, Milano, Italy).

For the pharmacological experiments, mexiletine (Sigma-Aldrich Merck Life Science, Milano, Italy) was daily dissolved in DMSO solutions. DMSO never exceeded 0.2%, a concentration without effect on Nav1.5 channels by itself. HEK 293 cells transfected with WT and mutant channels were incubated for 24h with mexiletine dissolved in the cell medium at the final concentration of 300µM. On the day of experiment, mexiletine was washed out prior to recording sodium currents.

Supplementary Figure S1

```
Nav1.1     GAIKSLRTLRALRPLRALSRFEGMRVVVNALLGAIPSIMNVLLVCLIFWL    1359
Nav1.2     GAIKSLRTLRALRPLRALSRFEGMRVVVNALLGAIPSIMNVLLVCLIFWL    1349
Nav1.3     GAIKSLRTLRALRPLRALSRFEGMRVVVNALVGAIPSIMNVLLVCLIFWL    1347
Nav1.4     GPIKSLRTLRALRPLRALSRFEGMRVVVNALLGAIPSIMNVLLVCLIFWL    1172
Nav1.5     GPIKSLRTLRALRPLRALSRFEGMRVVVNALVGAIPSIMNVLLVCLIFWL    1345
Nav1.6     GAIKSLRTLRALRPLRALSRFEGMRVVVNALVGAIPSIMNVLLVCLIFWL    1339
Nav1.7     GPIKSLRTLRALRPLRALSRFEGMRVVVNALIGAIPSIMNVLLVCLIFWL    1322
Nav1.8     APIKALRTLRALRPLRALSRFEGMRVVVDALVGAIPSIMNVLLVCLIFWL    1293
Nav1.9     MELKSFRTLRALRPLRALSQFEGMKVVVNALIGAIPAILNVLLVCLIFWL    1190

Nav1.1     LLLFLVMFIYAIFGMSNFAYVKREVGIDDMFNFETFGNSMICLFQITTSA    1724
Nav1.2     LLLFLVMFIYAIFGMSNFAYVKREVGIDDMFNFETFGNSMICLFQITTSA    1714
Nav1.3     LLLFLVMFIYAIFGMSNFAYVKKEAGIDDMFNFETFGNSMICLFQITTSA    1709
Nav1.4     LLLFLVMFIYSIFGMSNFAYVKKESGIDDMFNFETFGNSIICLFEITTSA    1536
Nav1.5     LLLFLVMFIYSIFGMANFAYVKWEAGIDDMFNFQTFANSMLCLFQITTSA    1711
Nav1.6     LLLFLVMFIFSIFGMSNFAYVKHEAGIDDMFNFETFGNSMICLFQITTSA    1705
Nav1.7     LLLFLVMFIYAIFGMSNFAYVKKEDGINDMFNFETFGNSMICLFQITTSA    1687
Nav1.8     LLLFLVMFIYSIFGMSSFPHVRWEAGIDDMFNFQTFANSMLCLFQITTSA    1661
Nav1.9     LLLFLIMFIYAILGMNWFSKVNPESGIDDIFNFKTFASSMLCLFQISTSA    1551
```

Figure S1. Amino acids alignment of Nav1.x proteins highlighting the position of the P1310L and Ins1687GR BrS mutations.

Supplementary Figure S2

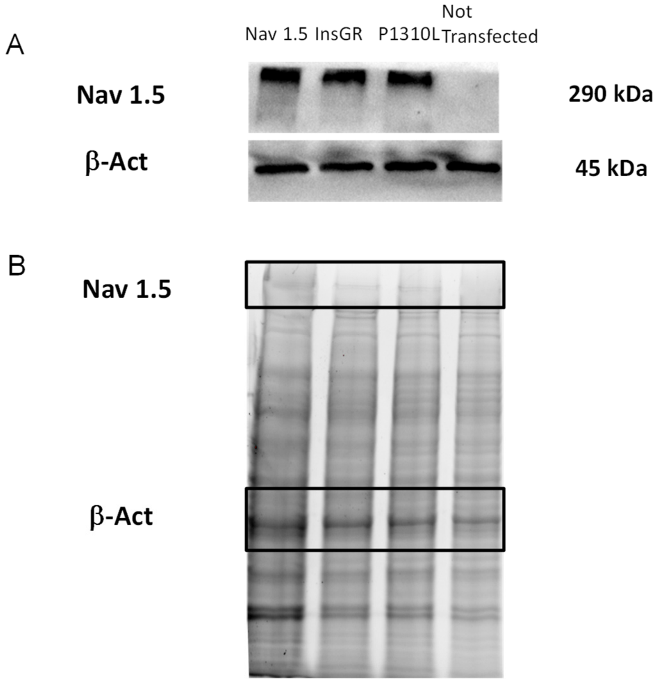

Figure S2. A) Immunoblotting analysis of Nav1.5 WT, Ins1687GR and P1310L channels expressed in HEK 293 cells as in Figure 7B. The positions of molecular weight markers are at the right of the blots. Expressions of β-actin are displayed as controls for the loaded protein amounts. B) Stain-free blot image, corresponding to the total protein load, of the immunoblot reported in A).

Supplementary References

1. Richards, S.; Aziz, N.; Bale, S.; Bick, D.; Das, S.; Gastier-Foster, J.; Grody, W.W.; Hegde, M.; Lyon, E.; Spector, E.; et al. Standards and guidelines for the interpretation of sequence variants: A joint consensus recommendation of the American College of Medical Genetics and Genomics and the Association for Molecular Pathology. *Genet. Med.* **2015**, *17*, 405–424, doi:10.1038/gim.2015.30.
2. Lieve, K.V.; Verkerk, A.O.; Podliesna, S.; van der Werf, C.; Tanck, M.W.; Hofman, N.; van Bergen, P.F.; Beekman, L.; Bezzina, C.R.; Wilde, A.A.M.; et al. Gain-of-function mutation in SCN5A causes ventricular arrhythmias and early onset atrial fibrillation. *Int. J. Cardiol.* **2017**, *236*, 187–193, doi:10.1016/j.ijcard.2017.01.113.
3. Maggi, L.; Ravaglia, S.; Farinato, A.; Brugnoni, R.; Altamura, C.; Imbrici, P.; Camerino, D.C.; Padovani, A.; Mantegazza, R.; Bernasconi, P.; et al. Coexistence of CLCN1 and SCN4A mutations in one family suffering from myotonia. *Neurogenetics* **2017**, *18*, 219–225, doi:10.1007/s10048-017-0525-5.
4. Portero, V.; Wilders, R.; Casini, S.; Charpentier, F.; Verkerk, A.O.; Remme, C.A. KV4.3 Expression Modulates NaV1.5 Sodium Current. *Front. Physiol.* **2018**, *9*, 178, doi:10.3389/fphys.2018.00178.
5. Attwell, D.; Cohen, I.; Eisner, D.; Ohba, M.; Ojeda, C. The steady state TTX-sensitive ("window") sodium current in cardiac Purkinje fibres. *Pflugers Arch.* **1979**, *379*, 137–142, doi:10.1007/BF00586939.
